# Plasma Sphingolipids as Potential Indicators of Hepatic Necroinflammation in Patients with Chronic Hepatitis C and Normal Alanine Aminotransferase Level

**DOI:** 10.1371/journal.pone.0095095

**Published:** 2014-04-15

**Authors:** Jun-Feng Li, Feng Qu, Su-Jun Zheng, Jin-Yu Ren, Hui-Li Wu, Mei Liu, Hui Liu, Feng Ren, Yu Chen, Jin-Lan Zhang, Zhong-Ping Duan

**Affiliations:** 1 The First Clinical Medical School, Lanzhou University, Lanzhou, China; 2 Artificial Liver Center, Beijing YouAn Hospital, Capital Medical University, Beijing, China; 3 State Key Laboratory of Bioactive Substance and Function of Natural Medicines, Institute of Materia Medica, Chinese Academy of Medical Sciences & Peking Union Medical College, Beijing, China; 4 Evergreen Wellness Center, Kansas College of Chinese Medicine, Wichita, Kansas, United States of America; 5 Department of Pathology, Beijing YouAn Hospital, Capital Medical University, Beijing, China; Saint Louis University, United States of America

## Abstract

Accurate estimation of hepatic necroinflammation caused by chronic hepatitis C (CHC) is crucial for prediction of prognosis and design of therapeutic strategy, which is particularly true for CHC patients with normal alanine aminotransferase (ALT) level. Recent studies have shown that sphingolipids have a close relationship with hepatitis C virus infection. The present study aimed to identify plasma sphingolipids related to hepatic necroinflammation. We included 120 treatment-naïve CHC patients and 64/120 had normal ALT levels (<40 U/L). CHC patients who underwent liver biopsies were subjected to Scheuer scoring analysis for scope of hepatic inflammation. Plasma sphingolipids were detected by high-performance liquid chromatography tandem mass spectrometry. Our results showed 44 plasma sphingolipids were detected altogether. Of all detected sphingolipids, hexosylceramide (HexCer) (d18∶1/22∶0) and HexCer (d18∶1/24∶0) showed a significant difference among G0/G1, G2, and G3/G4 (*P*<0.05). For identifying hepatic necroinflammation (G≥2), after adjusting other factors, the odds ratio (OR) of HexCer (d18∶1/22∶0) reached 1.01 (95% confidence interval [CI]: 1.00–1.02). Furthermore, the area under the curve (AUC) of HexCer (d18∶1/22∶0) was 0.7 (*P* = 0.01) and approached that of ALT (AUC = 0.78). However, in CHC patients with normal ALT, HexCer (d18∶1/22∶0) was an independent factor (OR: 1.02, 95% CI: 1.01–1.03) to identify the hepatic necroinflammation (G≥2). HexCer (d18∶1/22∶0) not only showed the largest AUC (0.78, *P* = 0.001), but also exhibited the highest specificity of all indicators. These results indicate that plasma HexCer (d18∶1/22∶0) is a potential indicator to distinguish hepatic necroinflammation in CHC patients. For CHC with normal ALT, the ability of HexCer (d18∶1/22∶0) to distinguish hepatic necroinflammation might be superior to conventional serum indicators.

## Introduction

Hepatitis C is a major public health problem, affecting over 2% of the global population who are at risk of cirrhosis or hepatocellular carcinoma [1], [2]. Persistent hepatitis C virus (HCV) infection triggers hepatic necroinflammation, which may eventually contribute to liver fibrogenesis [3]. It has been confirmed that chronic hepatitis C (CHC) patients with extensive periportal hepatocellular necrosis and inflammation have higher rates of fibrosis progression [4]. Therefore, the necroinflammatory activity should be regarded as a major clue for deciding therapy [5]. As the gold criterion for estimating hepatic necroinflammation, liver biopsies are recommended in treatment guidelines worldwide. Nevertheless, these biopsies are not undertaken widely nowadays because of the invasive procedures with potential complications [6]. Serum aminotransferases are used to evaluate hepatic injury indirectly, but are limited because 15–30% of CHC patients have consistently normal alanine aminotransferase (ALT) despite the presence of liver inflammation [7], [8]. Furthermore, through long-term follow-up, liver histological injury can also be found in CHC patients with normal serial ALT [9]. There is an urgent need to find novel noninvasive biomarkers to evaluate accurately hepatic necroinflammation in CHC patients, especially those with normal ALT, to identify candidates for antiviral therapy.

Sphingolipids are enriched in lipid rafts of biological membranes and provide a distribution platform for different signal processes including proliferation, differentiation and apoptosis [10],[11]. During infection, pathogens are able to exploit lipid rafts composed of sphingolipids and others to gain entry to their target host cells [12]. For HCV infection, it has been reported that, in HCV-infected chimeric mice, inhibition of serine palmitoyltransferase, which is a first-step enzyme in the sphingolipid biosynthetic pathway, could suppress replication of HCV [13]. Moreover, inhibitors of HCV replication can prevent the *de novo* synthesis of sphingolipids *in vitro* [14]. After persistent infection in CHC patients, altered plasma sphingosine 1-phosphate, as a bioactive sphingolipid, has also been found recently to be involved in liver fibrosis [15]. Thus, sphingolipids have a close relationship with CHC, especially for the metabolites ceramide, which have been implicated in apoptosis and necrosis of hepatic cells and contribute to progression of liver diseases [16], [17], [18].

We have previously established a mature high-performance liquid chromatography–tandem mass spectrometry (HPLC-MS/MS) method to quantify sphingolipids in other diseases [19]. Based on that improved quantitative high-throughput lipidomic platform, we initially found a relationship between plasma sphingolipids and hepatic inflammation in CHC [20]. However, the relationship between plasma sphingolipids and hepatic necroinflammation has only rarely been studied before, especially for CHC patients with normal ALT.

Therefore, in the present study, based on our previous improved HPLC-MS/MS methods and liver biopsy, we aimed to evaluate the diagnostic ability of plasma sphingolipids to identify hepatic necroinflammation (G≥2) among patients with CHC and the subgroup of patients with normal ALT levels, respectively.

## Materials and Methods

### Patients

**Figure 1:**
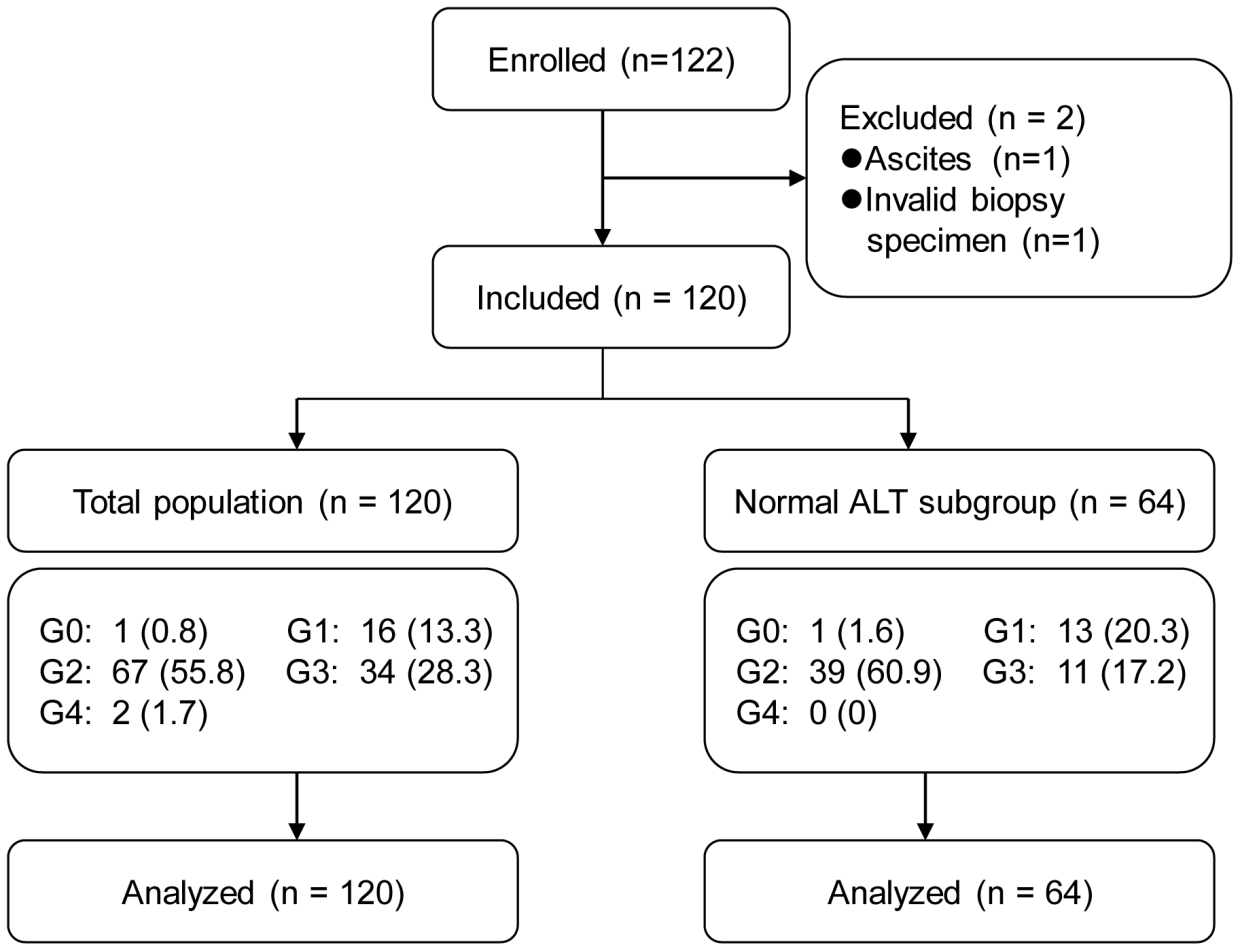
Flowchart of patient selection and study design. Serum ALT level <40 U/L was considered normal. Data are number of patients (%).

A group of 122 CHC patients with long-term follow-up, from Dingxi City, Gansu Province, China, were enrolled from July 2010 to June 2011. All the patients had a history of paid plasma donations with blood cell re-transfusions between 1992 and 1995, and did not receive antiviral treatment before being recruited into the present study. CHC diagnosis was made in accordance with established criteria [21], [22]. Patients with co-infections with other viruses or who were ineligible for liver biopsy were excluded. Two patients who were ineligible for liver biopsy, one due to ascites, the other due to the small size of the liver biopsy specimen, were excluded. One hundred and twenty patients were eligible for the study. To seek potential biomarkers among plasma sphingolipids for patients with normal ALT levels (<40 U/L), 64 patients were rescreened for analysis (Figure 1).

Fasting blood samples were collected on the day of the liver biopsy. Liver function and coagulation function tests were performed in all patients. Each patient gave signed informed consent at the beginning of the study. The study protocol was conducted in accordance with the provisions of the Declaration of Helsinki, 1975 and approved by the Institutional Review Board of Beijing YouAn Hospital, Capital Medical University, Beijing, China.

### Liver Biopsy

Liver biopsies were obtained from CHC patients under ultrasound guidance. Specimens were fixed in formalin and embedded in paraffin. Specimens longer than 1.5 cm, containing at least six complete portal areas, were reviewed by two senior pathologists who were unaware of the patients’ clinical data. The Scheuer scoring system was used to assess hepatic necroinflammatory activity, and G≥2 was considered to show the presence of necrosis [23]. Also, with fewer samples from G0 and G4 patients, G0 and G1, and G3 and G4 were respectively merged into G0/G1 or G3/G4 groups for statistical analysis.

### HPLC-MS/MS

Blood was collected into cold lithium heparin used as an anticoagulant and immediately centrifuged at 4°C at 8000 × *g*. The samples were stored at –80°C. All lipid standards were from Avanti Polar Lipids (Alabaster, AL, USA). Ultra Resi-analyzed grade methanol and HPLC grade methyl-tert-butyl ether (MTBE) were purchased from Mallinckrodt Baker (Phillipsburg, NJ, USA). Formic acid of analytical grade was obtained from TEDIA (Fairfield, OH, USA). Ammonium formate (purity >99.99%) was purchased from Sigma–Aldrich (St. Louis, MO, USA). Ultrapure water was prepared using a Milli-Q purification system (Millipore, Bedford, MA, USA). HPLC-MS/MS was performed on an Agilent 6410B Triple Quad mass spectrometer (QQQ; Agilent Technologies Santa Clara, CA, USA) comprising a triple quadrupole MS analyzer with an electrospray ionization interface and an Agilent 1200 RRLC system. Sphingolipidomic assays were performed at the Institute of Materia Medica, Peking Union Medical College (Beijing, China) as previously described [19].

### Statistical Analysis

Data were expressed as mean ± standard deviation (SD). In univariate analysis, depending on the data distribution according to the Kolmogorov–Smirnov test, continuous variables among multiple groups were analyzed by one-way analysis of variance or the nonparametric Kruskal–Wallis test. Comparisons between pairs of groups subsequently were analyzed by the least significant difference *t* test or Mann–Whitney test. Additionally, the independent-samples *t* test or Mann–Whitney test was used to compare G<2 against G≥2. Categoric variables were analyzed by Pearson χ^2^ test. Correlation analysis between plasma sphingolipids and inflammation grades was performed by Spearman’s rank correlation. Moving forward, the (LR) multivariate logistic regression analysis was performed, and the *P* values of entry and removal were respectively set to 0.05 and 0.1. The diagnostic value of each indicator with significant difference in univariate analysis was assessed by the area under the receiver operating characteristic (ROC) curve. Statistical analysis was performed using SPSS version 19.0 (Chicago, IL, USA), while a two-sided *P* value <0.05 was considered statistically significant.

## Results

### Clinical Characteristics of Patients

**Figure 2:**
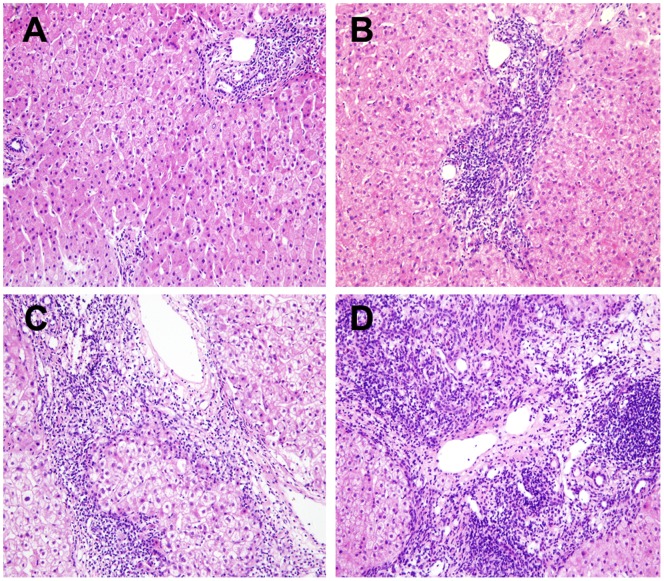
Liver histopathology of patients with G0/G1, G2 and G3/G4 according to the Scheuer scoring system. (A) G1, portal inflammation (HE ×200); (B) G2, mild piecemeal necrosis (HE ×200); (C) G3, moderate piecemeal necrosis (HE ×200); (D), G4, severe piecemeal necrosis and bridging necrosis (HE ×200).

The characteristics of the 120 patients who underwent a liver biopsy are summarized in Figure 1 and Table 1. The patient group comprised 57 (47.5%) male and 63 (52.5%) female patients with a mean age of 51.33 years. According to the Scheuer inflammation score, G2 was present in 67 patients, which accounted for the largest proportion (55.8%). This was followed by G3 (28.3%, 34/120) and G1 (13.3%, 16/120). G0 and G4 were found in 0.8% (1/120) and 1.7% (2/120) of the patients, respectively. In the subgroup of patients (n = 64) with normal ALT levels (<40 U/L), G2 predominated (39/64, 60.9%), followed by G1 (13/64, 20.3%) and G3 (11/64, 17.2%) (Figures 1 and 2). In univariate analysis of routine serological indicators in all patients, ALT (*P*<0.001), aspartate aminotransferase (AST) (*P*<0.001), γ-glutamyl transpeptidase (GGT) (*P*<0.001), total bile acid (TBA) (*P*<0.05), and prothrombin activity (PTA) (*P*<0.001) showed a significant difference among G0/G1, G2, and G3/G4 (Table 1).

**Table 1:** Characteristics of patients included and univariate analysis of routine indicators according to hepatic inflammation grade.

Variable	Total patients (n = 120)	G0/G1 (n = 17)	G2 (n = 67)	G3/G4 (n = 36)	*P* value
Age, yrs	51.33±7.33	50.41±8.42	50.88±7.07	52.58±7.33	0.36
Male, n (%)	57(47.50)	3 (17.65)	35 (52.24)	19 (52.78)	0.03
ALT (U/L)	60.42±70.88	29.34±17.66	48.37±33.78	97.52±112.35	<0.001
AST (U/L)	47.94±44.30	27.49±10.18	40.33±18.78	71.76±71.16	<0.001
TBIL (µmol/L)	16.51±7.25	13.40±4.47	17.06±7.42	16.96±7.75	0.13
DBIL (µmol/L)	3.26±1.36	2.89±1.14	3.23±1.23	3.50±1.65	0.43
TP (g/L)	70.93±4.95	69.84±3.65	71.23±5.27	70.89±4.89	0.59
Albumin (g/L)	43.21±2.36	44.34±1.84	43.19±2.38	42.72±2.43	0.06
Globulin (g/L)	29.19±17.29	26.08±3.57	30.50±22.84	28.23±4.19	0.09
GGT (U/L)	22.04±16.50	16.49±10.89	18.00±8.00	32.17±24.46	<0.001
ALP (U/L)	76.29±20.91	75.35±18.52	72.57±17.25	83.64±26.19	0.11
TBA (µmol/L)	11.24±46.76	4.57±2.75	13.70±62.44	9.80±6.61	0.004
Cholinesterase (U/L)	6957.09±1442.44	7541.44±2140.99	6882.53±1350.68	6819.92±1158.74	0.09
PTA (%)	92.41±9.11	98.47±6.30	92.96±9.39	88.50±7.99	<0.001

Data are means ± SD or number of patients (%).

*P* values are acquired by one-way analysis of variance or the nonparametric Kruskal-Wallis test for continuous variables depending on the data distribution, and by Pearson χ^2^ for categoric variables.

ALT: alanine aminotransferase; AST: aspartate aminotransferase; TBIL: total bilirubin; DBIL: direct bilirubin; TP: total protein; GGT: γ-glutamyl transpeptidase; ALP, alkaline phosphatase; TBA: total bile acid; PTA: prothrombin activity.

### Relationship between Plasma Sphingolipids and Hepatic Inflammation Grades in CHC Patients

**Figure 3:**
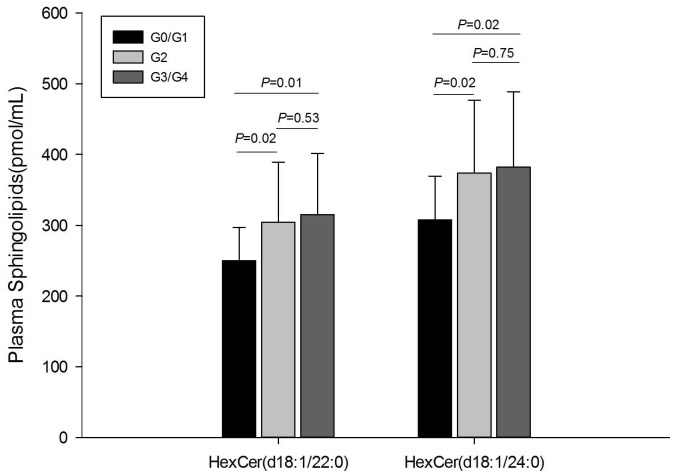
Plasma sphingolipids with significant difference by hepatic inflammation grades in patients with CHC.

Plasma sphingolipids in the 120 patients were analyzed by HPLC-MS/MS, and 44 sphingolipids were identified and quantified (Table S1). Two plasma sphingolipids showed a significant difference among G0/G1, G2, and G3/G4 (*P*<0.05). hexosylceramide (HexCer) (d18∶1/22∶0), which differed significantly between G0/G1 and G2 (*P* = 0.02), also showed a significant difference between G0/G1 and G3/G4 (*P* = 0.01). Similarly, another sphingolipid HexCer (d18∶1/24∶0) showed a significant difference in both G2 versus G0/G1 (*P* = 0.02) and G3/G4 versus G0/G1 (*P* = 0.02) (Figure 3). To analyze further the correlation between these two sphingolipids and hepatic inflammation grades, Spearman’s correlation analysis was performed. The results revealed that HexCer (d18∶1/22∶0) correlated with inflammation grades, and the correlation coefficient (r) was 0.21 (*P* = 0.02). HexCer (d18∶1/24∶0) also showed a correlation with inflammation grade (r = 0.19, *P* = 0.04).

### Distinction of Hepatic Necroinflammation (G≥2) in CHC Patients

Hepatic necroinflammatory activity is one of the most important predictors of fibrosis progression in CHC patients [4], [5], [24]. Likewise, the identification of hepatic necroinflammation (G≥2) is essential for assessment of disease status in CHC patients. We analyzed plasma sphingolipids and routine clinical indicators to establish better noninvasive markers. Of all plasma sphingolipids, ceramide (Cer) (d18∶1/18∶1), HexCer (d18∶1/16∶0), HexCer (d18∶1/22∶0), HexCer (d18∶1/24∶1), and HexCer (d18∶1/24∶0) showed a significant difference between G<2 and G≥2 (*P*<0.05) in univariate analysis. Routine serological indicators such as ALT, AST, albumin, globulin, GGT, TBA, cholinesterase, and PTA also showed significant differences between G<2 and G≥2 (*P*<0.05). To determine independent factors for the absence or presence of necroinflammation (G<2 versus. G≥2), the multivariate logistic regression analysis was performed. Sex, ALT, AST, GGT, cholinesterase, PTA, Cer (d18∶1/18∶1), HexCer (d18∶1/16∶0), HexCer (d18∶1/22∶0), HexCer (d18∶1/24∶1), and HexCer (d18∶1/24∶0) were initially included in multivariate analysis. The final results showed that the odds ratio (OR) for HexCer (d18∶1/22∶0) reached 1.01 [95% confidence interval (CI): 1.0–1.02]. In addition, the OR of AST and PTA was 1.07 (95% CI: 1.01–1.13) and 0.89 (95% CI: 0.81–0.98), respectively (Table 2).

**Table 2:** Indicators related to necroinflammation in CHC patients in univariate and multivariate analyses.

Variable	G<2 (n = 17)	G≥2 (n = 103)	*P* value	OR (95% CI)
Age, yrs	50.41±8.42	51.48±7.17	0.83	
Male, n (%)	3 (17.65)	54 (52.43)	0.01	
ALT (U/L)	29.34±17.66	65.55±75.00	<0.001	
AST (U/L)	27.49±10.18	51.32±46.83	<0.001	1.07 (1.01–1.13)
TBIL (µmol/L)	13.40±4.47	17.02±7.50	0.05	
DBIL (µmol/L)	2.89±1.14	3.32±1.39	0.21	
TP (g/L)	69.84±3.65	71.11±5.12	0.33	
Albumin (g/L)	44.34±1.84	43.02±2.40	0.03	
Globulin (g/L)	26.08±3.57	29.70±18.57	0.03	
GGT (U/L)	16.49±10.89	22.95±17.12	0.01	
ALP (U/L)	75.35±18.52	76.44±21.36	0.58	
TBA (µmol/L)	4.57±2.75	12.34±50.41	0.04	
Cholinesterase (U/L)	7541.44±2140.99	6860.64±1281.44	0.03	
PTA (%)	98.47±6.30	91.40±9.14	0.002	0.89 (0.81–0.98)
Cer(d18∶1/18∶1) (pmol/mL)	3.85±1.11	3.30±1.33	0.03	
HexCer(d18∶1/16∶0) (pmol/mL)	1131.87±525.58	1324.62±493.48	0.03	
HexCer(d18∶1/22∶0) (pmol/mL)	250.11±46.82	308.01±85.18	0.01	1.01 (1.00–1.02)
HexCer(d18∶1/24∶1) (pmol/mL)	372.25±106.73	483.20±175.46	0.001	
HexCer(d18∶1/24∶0) (pmol/mL)	307.86±61.45	376.58±103.94	0.01	

Data are means ± SD or number of patients (%).

*P* values are acquired by independent-samples *t* test or the Mann-Whitney test for continuous variables depending on the data distribution, and by Pearson χ^2^ for categoric variables.

OR: odds ratio; CI: confidence interval; ALT: alanine aminotransferase; AST: aspartate aminotransferase; TBIL: total bilirubin; DBIL: direct bilirubin; TP: total protein; GGT: γ-glutamyl transpeptidase; ALP, alkaline phosphatase; TBA: total bile acid; PTA: prothrombin activity; Cer: ceramide; HexCer: hexosylceramide.

Subsequently, the ROC analysis was performed to determine the diagnostic value of these significantly altered indicators. The area under curve (AUC) of HexCer (d18∶1/22∶0) reached 0.7 (*P* = 0.01). Although it was lower than that of ALT (AUC = 0.78, *P*<0.001), the specificity and positive likelihood ratio of HexCer (d18∶1/22∶0) (0.82 and 3.30, respectively) were higher than the routine serological indicators, including ALT. The AUC value of the other plasma sphingolipids HexCer (d18∶1/24∶0), HexCer (d18∶1/24∶1) ranged from 0.69 to 0.70 (all *P*<0.05); both of which were larger than that of globulin, cholinesterase, albumin, and TBA (Table S2).

### Identification of Hepatic Necroinflammation (G≥2) in the Subgroup of CHC Patients with Normal ALT levels

In order to evaluate whether sphingolipids could differentiate the hepatic necroinflammation (G≥2) in the subgroup of CHC patients with normal ALT levels, we also performed a univariate and multivariate analysis (Table 3). Routine serological indicators AST, total bilirubin, albumin, GGT, and PTA were significantly different between G<2 and G≥2 (*P*<0.05). HexCer (d18∶1/22∶0), HexCer (d18∶1/24∶1), and HexCer (d18∶1/24∶0) showed significant differences between G<2 and G≥2 (*P*<0.05) among all the plasma sphingolipids. During the logistic regression analysis, sex, AST, total bilirubin, GGT, PTA, HexCer (d18∶1/22∶0), HexCer (d18∶1/24∶1), and HexCer (d18∶1/24∶0) were included. The final multivariate results revealed that HexCer (d18∶1/22∶0) was independent of other factors and its OR value was 1.02 (95% CI: 1.01–1.03).

**Table 3:** Indicators related to necroinflammation (G≥2) in CHC patients with normal ALT in univariate and multivariate analyses.

Variable	G<2 (n = 14)	G≥2 (n = 50)	*P* value	OR (95% CI)
Age, yrs	49.29±8.84	51.84±6.97	0.26	
Male, n (%)	1(7.14)	18(36.00)	0.08	
AST (U/L)	24.13±7.44	30.86±8.61	0.01	
TBIL (µmol/L)	12.53±4.15	17.48±8.40	0.02	
DBIL (µmol/L)	2.67±1.10	3.24±1.36	0.11	
TP (g/L)	70.24±3.32	71.10±5.39	0.58	
Albumin (g/L)	44.60±1.83	43.17±2.36	0.04	
Globulin (g/L)	26.35±3.53	27.98±4.23	0.19	
GGT (U/L)	12.97±4.27	16.61±7.31	0.03	
ALP (U/L)	74.98±20.09	71.10±13.91	0.41	
TBA (µmol/L)	4.21±2.43	6.54±4.72	0.10	
Cholinesterase (U/L)	7546.39±2232.91	6884.89±1437.77	0.19	
PTA (%)	99.59±6.18	93.39±10.04	0.02	
HexCer(d18∶1/16∶0) (pmol/mL)	1079.01±495.41	1271.33±390.77	0.05	
HexCer(d18∶1/22∶0) (pmol/mL)	236.83±40.12	315.53±90.33	<0.001	1.02(1.01–1.03)
HexCer(d18∶1/24∶1) (pmol/mL)	340.53±86.46	490.90±189.20	<0.001	
HexCer(d18∶1/24∶0) (pmol/mL)	291.15±50.55	377.49±102.57	<0.001	
dhCer(d18∶0/24∶1) (pmol/mL)	35.10±11.78	48.48±22.56	0.05	

Data are means ± SD or number of patients (%).

*P* values are acquired by independent-samples *t* test or the Mann-Whitney test for continuous variables, and by Pearson χ^2^ for categoric variables.

OR: odds ratio; CI: confidence interval; AST: aspartate aminotransferase; TBIL: total bilirubin; DBIL: direct bilirubin; TP: total protein; GGT: γ-glutamyl transpeptidase; ALP, alkaline phosphatase; TBA: total bile acid; PTA: prothrombin activity; Cer: ceramide; HexCer: hexosylceramide; dhCer: dihydroceramide.

In the ROC analysis, two plasma sphingolipids demonstrated larger AUCs than those of routine serological indicators. Of concern was the fact that HexCer (d18∶1/22∶0) with the largest AUC (0.78, *P* = 0.001) exhibited the highest specificity (1.0) when the optimal cutoff value was set to 281.37 pmol/mL. The AUC of HexCer (d18∶1/24∶1) followed closely, which reached 0.75 (95% CI: 0.62–0.87, *P*<0.05) and was the same as AST. The AUCs of total bilirubin, PTA, and GGT fluctuated from 0.69 to 0.71 (Table 4).

**Table 4:** ROC analysis of indicators related to identification of necroinflammation (G≥2) in CHC patients with normal ALT levels.

Variable	AUC(95%CI)	*P* value	Cut-off value	Se	Sp	PLR	NLR
HexCer(d18∶1/22∶0)	0.78(0.67–0.90)	0.001	281.37	0.62	1.00	–	0.38
HexCer(d18∶1/24∶1)	0.75(0.62–0.87)	0.01	452.10	0.56	1.00	–	0.44
AST	0.75(0.60–0.91)	0.004	27.15	0.74	0.79	3.52	0.33
HexCer(d18∶1/24∶0)	0.74(0.62–0.87)	0.01	371.63	0.52	1.00	–	0.48
TBIL	0.71(0.56–0.87)	0.02	11.55	0.80	0.64	2.24	0.31
PTA	0.71(0.56–0.86)	0.02	96.10	0.64	0.79	2.99	0.46
GGT	0.69(0.54–0.84)	0.03	14.20	0.52	0.86	3.64	0.56

The indicators whose *P* values of AUC were less than 0.05 were listed in the Table.

AUC: area under curve; CI: confidence interval; Se: sensitivity; Sp: specificity; PLR: positive likelihood ratio; NLR: negative likelihood ratio; HexCer: hexosylceramide; AST: aspartate aminotransferase; TBIL: total bilirubin; PTA: prothrombin activity; GGT: γ-glutamyl transpeptidase.

## Discussion

In our cohort, all CHC patients had definite histories of plasma donation with blood cell re-transfusion and did not receive antiviral treatment before enrollment. Therefore, in this context of an interferon-naïve state of disease, we were able to obtain true results of sphingolipids changes after eliminating the influence of interferon therapy on hepatic inflammation. From our data, for the first time, we reported that HexCer (d18∶1/22∶0) might be a surrogate marker for assessing hepatic necroinflammation in patients with CHC, especially, for those with normal ALT level.

Currently, although increasing evidence confirms the close relationship between sphingolipids and liver diseases [18], for example, ceramides which interact with several pathways involved in oxidative stress, inflammation and apoptosis, can promote the progress of nonalcoholic fatty liver disease [25], the role of sphingolipids in hepatic inflammation in CHC patients remains to be elucidated. In our study, the univariate and multivariate analyses showed that HexCer (d18∶1/22∶0) correlated with alteration of hepatic inflammation grade in CHC, and verified that HexCer (d18∶1/22∶0) had an independent relationship with hepatic necroinflammation (G≥2) after adjusting for other factors, especially in CHC patients with normal ALT level. Our results show that HexCer (d18∶1/22∶0), which was elevated in CHC patients with hepatic necroinflammation, might contribute uniquely to the pathogenesis of hepatic necroinflammation in CHC. Although there is no direct evidence of the role of HexCer (d18∶1/22∶0) in hepatic necroinflammation caused by CHC, there are several possible mechanisms that may be involved in the relationship between this specific sphingolipid and hepatic necroinflammation. One *in vivo* study has reported that α-galactosylceramide induces pronounced cytokine responses that could contribute to hepatic inflammation, by activating natural killer T cells [26]. In addition, α-galactosylceramide can also lead to hepatic injury through tumor necrosis factor, which increased Fas-ligand expression of natural killer T cells in an animal model [27]. However, experimental studies are needed to determine the exact underlying mechanisms responsible for the association of HexCer (d18∶1/22∶0) with hepatic necroinflammation in CHC.

Furthermore, we focused on the potential of plasma sphingolipids to identify the presence of hepatic necroinflammation (G≥2) in CHC. Another interesting finding in our study was that, although the AUC value of these sphingolipids to identify hepatic necroinflammation (G≥2) was not larger than that of routine serological indicators in the total patient population, others such as negative likelihood ratio and positive likelihood ratio of some sphingolipids were better indicators than routine serological indicators. This indicated that plasma sphingolipids, especially HexCer (d18∶1/22∶0), which was independently linked to hepatic necroinflammation with the largest AUC of all sphingolipids, had a certain superiority in identifying hepatic necroinflammation in CHC patients.

We also verified the superiority of plasma sphingolipids compared with routine serological indicators to reflect the presence of necroinflammation (G≥2) in CHC patients with normal ALT levels. At present, elevated ALT level, as an indirect surrogate marker for hepatic necrosis, still has poor ability to identify hepatic inflammation in CHC patients [28], which is particularly true for CHC patients with normal ALT and liver inflammation [7], [8]. Thus, it is more meaningful to find new markers for hepatic necroinflammation in CHC patients with normal ALT for deciding therapeutic strategy. Our results revealed that plasma HexCer (d18∶1/22∶0) exhibited the largest AUC (0.78, *P* = 0.001) and the highest specificity (1.0) of all the indicators including plasma sphingolipids and routine serological indicators. This suggests that HexCer (d18∶1/22∶0) might become a potential marker for identifying hepatic necroinflammation in CHC patients with normal ALT.

Although HexCer (d18∶1/24∶0) showed that its AUC approached that of HexCer (d18∶1/22∶0) in ROC analysis, HexCer (d18∶1/24∶0) was not an independent factor to identify hepatic necroinflammation in CHC in multivariate analysis. However, we speculated that the association with small samples and poor statistical power could not be ruled out. We confirmed the superior diagnostic value of HexCer (d18∶1/22∶0) to identify hepatic necroinflammation in patients with CHC and normal ALT level.

There were some limitations in our study. We analyzed the relationship between the plasma sphingolipids and hepatic necroinflammation based on a long-term (∼20 years) follow-up in a cohort of interferon-naïve CHC patients, and the actual number of cases was not large, and the different inflammation groups were not matched exactly for sex. However, by adjusting for sex, the multivariate analysis showed that sex had minimal effects on plasma sphingolipid level.

In summary, based on liver biopsies and HPLC-MS/MS, we have found for the first time that plasma sphingolipid HexCer (d18∶1/22∶0) has a positive diagnostic value to distinguish hepatic necroinflammation in CHC, especially in patients with normal ALT levels. Meanwhile, our discovery of plasma sphingolipids has opened new directions of research to identify novel noninvasive markers for progression of CHC.

## Supporting Information

Table S1 **Plasma sphingolipids profile of untreated chronic hepatitis C patients with G0/G1, G2, G3/G4.** Data are expressed as mean ± standard deviation, *P* values were calculated by one-way analysis of variance or the nonparametric Kruskal-Wallis test.
(DOC)

Table S2 **Performance of indicators for identification of liver necroinflammation (G≥2) in ROC analysis.** The indicators whose *P* values of AUC were less than 0.05 are listed in the Table.
(DOC)
