# Supplementary material for: Plasma Sphingolipids as Potential Indicators of Hepatic Necroinflammation in Patients with Chronic Hepatitis C and Normal Alanine Aminotransferase Level
Source: PLoS One. 2014 Apr 15;9(4):e95095. doi: 10.1371/journal.pone.0095095 (PMC3988168; doi:10.1371/journal.pone.0095095)
Supplement: Table S1 — Plasma sphingolipids profile of untreated chronic hepatitis C patients with G0/G1, G2, G3/G4. Data are expressed as mean ± standard deviation, P values were calculated by one-way analysis of variance or the nonparametric Kruskal-Wallis test. (DOC) [file pone.0095095.s001.doc]

**Table S1. Plasma sphingolipids profile of untreated chronic hepatitis C patients with G0/G1, G2, G3/G4.**

| **Variable (pmol/mL)** | **G0/G1(n=17)** | **G2(n=67)** | **G3/G4(n=36)** | ***P* value** |
| --- | --- | --- | --- | --- |
| Sphingosine-1-P | 138.09±34.97 | 146.82±37.28 | 152.67±40.19 | 0.42 |
| Cer(d18:1/16:0)-1-P | 24.85±10.26 | 26.29±9.98 | 26.49 ±11.97 | 0.86 |
| Cer(d18:1/18:1)-1-P | 14.45±10.89 | 16.75±13.82 | 17.70 ±13.71 | 0.71 |
| Cer(d18:1/18:0)-1-P | 222.24±54.91 | 211.61±81.92 | 214.95 ±86.19 | 0.52 |
| Cer(d18:1/20:0)-1-P | 22.00±10.79 | 20.23±11.92 | 21.39 ±11.92 | 0.66 |
| Cer(d18:1/24:0)-1-P | 12.56±5.53 | 14.39±5.08 | 14.92 ±6.34 | 0.36 |
| Cer(d18:1/16:0) | 129.98±33.10 | 138.98±41.26 | 134.12 ±36.48 | 0.90 |
| Cer(d18:1/18:1) | 3.85±1.11 | 3.43±1.45 | 3.08±1.07 | 0.05 |
| Cer(d18:1/18:0) | 29.24±11.89 | 27.77±15.00 | 26.20±11.69 | 0.64 |
| Cer(d18:1/20:0) | 22.44±11.18 | 20.99±7.57 | 20.99±9.44 | 0.88 |
| Cer(d18:1/22:0) | 175.48±54.10 | 174.88±82.60 | 163.21±85.97 | 0.27 |
| Cer(d18:1/24:1) | 401.28±116.97 | 420.79±159.15 | 380.38±119.38 | 0.55 |
| Cer(d18:1/24:0) | 586.72±107.29 | 583.19±116.43 | 565.19±116.73 | 0.71 |
| Cer(d18:1/26:0) | 4.37±1.85 | 4.74±2.33 | 4.34±1.75 | 0.94 |
| dhSphingosine | 2.84±1.47 | 3.23±2.13 | 2.89±1.77 | 0.85 |
| dhSphingosine-1-P | 24.21±6.75 | 25.57±6.34 | 26.07±5.55 | 0.59 |
| HexCer(d18:1/6:0) | 5.63±6.33 | 5.03±4.47 | 3.26±1.87 | 0.22 |
| HexCer(d18:1/12:0) | 14.74±7.94 | 16.38±15.31 | 18.92±15.98 | 0.49 |
| HexCer(d18:1/14:0) | 7.10±4.03 | 7.33±2.83 | 7.63±2.91 | 0.57 |
| HexCer(d18:1/16:0) | 1131.87±525.58 | 1309.16±453.83 | 1353.39±565.70 | 0.10 |
| HexCer(d18:1/18:1) | 12.67±5.51 | 12.73±6.74 | 12.01±6.00 | 0.86 |
| HexCer(d18:1/18:0) | 10.12±4.86 | 11.18±6.64 | 11.03±6.06 | 0.95 |
| HexCer(d18:1/20:0) | 18.69±11.45 | 19.71±12.48 | 20.16±12.40 | 0.75 |
| HexCer(d18:1/22:0) | 250.11±46.82 | 304.32±84.89 | 314.89±86.50 | **0.02** |
| HexCer(d18:1/24:1) | 372.25±106.73 | 481.21±179.75 | 486.92±169.63 | 0.05 |
| HexCer(d18:1/24:0) | 307.86±61.45 | 373.62±103.21 | 382.08±106.54 | **0.03** |
| dhCer(d18:0/16:0) | 7.66±1.67 | 8.20±2.81 | 8.07±2.49 | 0.98 |
| dhCer(d18:0/18:0) | 7.41±2.38 | 7.37±2.90 | 7.59±2.98 | 0.79 |
| dhCer(d18:0/20:0) | 35.05±15.00 | 37.45±16.98 | 37.32±17.02 | 0.96 |
| dhCer(d18:0/22:0) | 128.27±59.07 | 134.79±79.05 | 127.74±60.07 | 1.00 |
| dhCer(d18:0/24:1) | 38.56±13.52 | 47.67±21.99 | 50.28±30.10 | 0.37 |
| dhCer(d18:0/24:0) | 106.36±47.04 | 121.79±59.54 | 124.32±54.28 | 0.57 |
| dhCer(d18:0/26:1) | 5.26±2.37 | 6.78±5.00 | 6.33±2.59 | 0.52 |
| dhCer(d18:0/26:0) | 8.85±3.86 | 10.29±5.20 | 9.56±3.79 | 0.82 |
| Lyso-SM(d18:1) | 5.29±1.58 | 5.34±1.88 | 5.40 ±1.83 | 0.97 |
| SM(d18:1/12:0) | 10.48±2.85 | 9.84±2.89 | 10.69±2.88 | 0.47 |
| SM(d18:1/14:0) | 4.75±1.11 | 4.74±0.97 | 4.93±1.14 | 0.81 |
| SM(d18:1/16:0) | 3354.20±434.08 | 3415.04±398.06 | 3395.43±337.34 | 0.84 |
| **Table S1. cont.** | | | | |
| SM(d18:1/18:1) | 791.90±214.15 | 702.78±198.29 | 692.83±217.89 | 0.23 |
| SM(d18:1/18:0) | 1524.38±184.71 | 1503.93±236.16 | 1501.07±196.03 | 0.93 |
| SM(d18:1/20:0) | 18.79±2.86 | 18.40±2.93 | 18.21±2.81 | 0.49 |
| SM(d18:1/22:0) | 28.25±6.08 | 26.38±4.02 | 26.55±4.53 | 0.31 |
| SM(d18:1/24:1) | 2903.30±701.51 | 2988.99±711.33 | 2957.17±644.09 | 0.93 |
| SM(d18:1/24:0) | 1631.00±333.24 | 1598.16±224.71 | 1572.72±244.82 | 0.72 |

Data are expressed as mean ± standard deviation, *P* values were calculated by one-way analysis of variance or the nonparametric Kruskal-Wallis test.

Cer: ceramide; dhSphingosine: dihydrosphingosine; HexCer: hexosylceramide; dhCer: dihydroceramide; SM: sphingomyelin
