# Supplementary material for: Plasma Sphingolipids as Potential Indicators of Hepatic Necroinflammation in Patients with Chronic Hepatitis C and Normal Alanine Aminotransferase Level
Source: PLoS One. 2014 Apr 15;9(4):e95095. doi: 10.1371/journal.pone.0095095 (PMC3988168; doi:10.1371/journal.pone.0095095)
Supplement: Table S2 — Performance of indicators for identification of liver necroinflammation (G≥2) in ROC analysis. The indicators whose P values of AUC were less than 0.05 are listed in the Table. (DOC) [file pone.0095095.s002.doc]

**Table S2. Performance of indicators for identification of liver necroinflammation (G ≥2) in ROC analysis**

| **Variable** | **AUC(95%CI)** | ***P* value** | **Cut-off value** | **Se** | **Sp** | **PLR** | **NLR** |
| --- | --- | --- | --- | --- | --- | --- | --- |
| ALT | 0.78(0.65-0.90) | <0.001 | 29.05 | 0.83 | 0.65 | 2.34 | 0.27 |
| AST | 0.77(0.65-0.89) | <0.001 | 27.15 | 0.85 | 0.65 | 2.42 | 0.23 |
| PTA | 0.74(0.62-0.86) | 0.002 | 96.10 | 0.71 | 0.73 | 2.60 | 0.40 |
| GGT | 0.71(0.56-0.85) | 0.01 | 14.05 | 0.67 | 0.76 | 2.85 | 0.43 |
| HexCer(d18:1/22:0) | 0.70(0.59-0.81) | 0.01 | 279.68 | 0.58 | 0.82 | 3.30 | 0.51 |
| HexCer(d18:1/24:0) | 0.70(0.59-0.81) | 0.01 | 389.51 | 0.44 | 0.94 | 7.43 | 0.60 |
| HexCer(d18:1/24:1) | 0.69(0.57-0.80) | 0.01 | 465.96 | 0.50 | 0.88 | 4.29 | 0.56 |
| Cer(d18:1/18:1) | 0.67(0.54-0.80) | 0.02 | 2.46 | 1.00 | 0.32 | 1.47 | 0.00 |
| Globulin | 0.67(0.53-0.80) | 0.03 | 24.65 | 0.83 | 0.47 | 1.58 | 0.35 |
| Cholinesterase | 0.66(0.51-0.82) | 0.03 | 8740.00 | 0.35 | 0.94 | 6.06 | 0.69 |
| HexCer(d18:1/16:0) | 0.66(0.50-0.83) | 0.03 | 844.79 | 0.90 | 0.47 | 1.71 | 0.21 |
| Albumin | 0.66(0.54-0.78) | 0.04 | 43.45 | 0.76 | 0.56 | 1.75 | 0.42 |
| TBA | 0.66(0.53-0.79) | 0.04 | 5.00 | 0.57 | 0.76 | 2.43 | 0.56 |

The indicators whose *P* values of AUC were less than 0.05 are listed in the Table.

AUC: area under the curve; 95% CI: 95% confidence interval; Se: sensitivity; Sp: specificity; PLR: positive likelihood ratio; NLR: negative likelihood ratio. ALT: alanine aminotransferase; AST: aspartate aminotransferase; PTA: prothrombin activity; GGT: γ-glutamyl transpeptidase; HexCer: hexosylceramide; Cer: ceramide; TBA: total bile acid
